# Sequential Response of Sage Antioxidant Metabolism to Chilling Treatment

**DOI:** 10.3390/molecules24224087

**Published:** 2019-11-12

**Authors:** Andrzej Kalisz, Agnieszka Sękara, Robert Pokluda, Aleš Jezdinský, Jarmila Neugebauerová, Katalin Angéla Slezák, Edward Kunicki

**Affiliations:** 1 Department of Horticulture, University of Agriculture in Krakow, 29-Listopada 54, 31-425 Kraków, Poland; agnieszka.sekara@urk.edu.pl (A.S.); edward.kunicki@urk.edu.pl (E.K.); 2 Department of Vegetable Sciences and Floriculture, Mendel University in Brno, Valtická 337, 691 44 Lednice, Czech Republic; robert.pokluda@mendelu.cz (R.P.); ales.jezdinsky@mendelu.cz (A.J.); jarmila.neugebauerova@mendelu.cz (J.N.); 3 Department of Vegetable and Mushroom Growing, Szent István University, Villányi 29-43, 1118 Budapest, Hungary; Slezak.Katalin@kertk.szie.hu

**Keywords:** antioxidants, chilling stress, cultivars, reactive oxygen species, *Salvia officinalis* L.

## Abstract

Chilling influences the growth and metabolism of plants. The physiological response and acclimatization of genotypes in relation to stress stimulus can be different. Two sage cultivars: ‘Icterina’ and ‘Purpurascens’ were subjected to 4 °C and 18 °C (control), and sampled between the 5th and 14th day of the treatment. Ascorbate peroxidase (APX) activity was up-regulated in chilled ‘Purpurascens’ on the 14th day, while guaiacol peroxidase (GPX) activity increased on the 10th and 12th day in relation to the control. GPX activity of the control ‘Icterina’ was frequently higher than chilled plants, and chilling did not affect APX activity of that cultivar. Catalase activity remained stable in both sage cultivars. Chilled ‘Purpurascens’ showed a significant increase in total phenolics contents on the 5th, 7th, and 12th day and in total antioxidant capacity on the 5th and 10th day as compared to the control for respective sampling days. Higher malondialdehyde content was found in chilled plants on the 12th, or 14th day, differences reached 26–28% of the controls. Chilling caused significant decrease in dry matter content. The stress response was more stable and effective in ‘Icterina’, while more dynamic changes were found for ‘Purpurascens’. Based on our results, we propose to use ‘Purpurascens’ for targeted stress-induced studies and ‘Icterina’ for field applications.

## 1. Introduction

Chilling is one of the major abiotic stresses influencing the growth and development of crops and their metabolism. The equilibrium between production and scavenging of reactive oxygen species (ROS) may be disturbed by a number of abiotic factors, which may increase the intracellular levels of ROS [1,2]. It is known that ROS may cause oxidative damage in plant cells [3]. The primary targets of ROS are unsaturated lipids, giving in to oxidation and the increased synthesis of the lipid oxidation by-product—malondialdehyde (MDA) [4]. Plants developed defense mechanisms against oxidative bursts, that are activated during stress, to maintain ROS at non-toxic levels [5]. These mechanisms include the activation of antioxidant enzymes and the production of several non-enzymatic antioxidative compounds [2,6,7,8,9].

The patterns of activity of antioxidant enzymes under chilling can be different [10]. Lee and Lee [11] observed a significant increase in the activity of the superoxide dismutase (SOD) and enzymes of ascorbate-glutathione (AsA-GSH) cycle but catalase (CAT) was deactivated in the leaves of cucumber under chilling. Airaki et al. [12] showed the induction of ascorbate peroxidase (APX), CAT, and monodehydroascorbate reductase (MDAR) after one day of low temperature treated sweet pepper, but such induction was not noted for glutathione reductase (GR). During the next day, the activity of CAT remained stable, while GR activity was reduced by almost half. Cheng et al. [13] showed that the activities of guaiacol peroxidase, catalase, and ascorbate peroxidase significantly increased on the 3rd day of chilling treatment in watermelon plants, and the activities of guaiacol peroxidase and catalase declined to basal levels on 7th day of chilling treatment. Moreover, APX activity remained elevated in the next four days. On the other hand, there are scientific reports of a similar time of induction of enzyme activities under stress, although the intensity of these changes may vary [14]. The response of non-enzymatic antioxidants to chilling stress is observed usually after a strictly specified time [9,15,16]. Cheng et al. [13] described similar patterns of glutathione and ascorbate concentration changes in watermelon plants up to the 3rd day of chilling, but later, the ascorbate content remained stable, while the glutathione content increased until the 7th day. Sivaci et al. [17] showed an increase in ascorbic acid and total phenolics content between the 5th and 10th day of chilling of pepino, while the level of carotenoid compounds decreased on the 5th day in comparison to control plants and then their concentration was similar to the control. Proietti et al. [18] did not find significant changes in ascorbate content between chilled and control spinach on the 2nd day of chilling, but later it started to rise and reached the maximum after seven days, with a 41% increase with respect to control leaves.

There is also a difference in the response of antioxidant mechanisms to stress among plant genotypes [8,19,20]. Kalisz et al. [15] showed the response of six basil cultivars to low temperature and found an increase of L-ascorbic acid in most tested basil cultivars, but total phenolic content increased significantly only in the lettuce leaf basil. Xu et al. [21] noted that cucumber cultivars varied with respect to CAT, guaiacol peroxidase (GPX), and GR activity but not SOD activity under low temperature. This shows how important it is to evaluate the response of different crop cultivars to abiotic stress because higher activities of antioxidant enzymes and higher content of non-enzymatic antioxidants under stress are associated with genotype-dependent tolerance to chilling [8]. The aim of our study on sage cultivars was to evaluate (1) how enzyme activity changes over time, assuming that it will vary depending on the enzyme type; (2) changes in total phenolics content and total antioxidant activity assuming that such changes will be visible in time showing activation of non-enzymatic antioxidant mechanisms; (3) if genotype differences will occur in response to chilling in tested sage cultivars.

## 2. Results and Discussion

**Figure 1:**
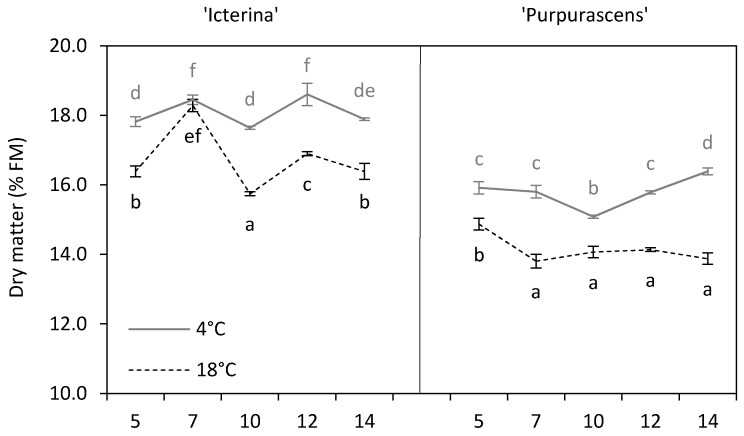
Dry matter (DM) of ‘Icterina’ and ‘Purpurascens’ sage cultivars at different time points. Control plants (black dashed line) were grown at 18 °C, and chilled plants (grey line) were treated at 4 °C. Each graph is labeled with the number of the day (days from the start of chilling). Error bars represent ± standard deviation (*n* = 3). Different letters for the particular sage cultivar indicate significant differences in DM content as affected by the chilling temperature and sampling time according to the Tukey’s HSD test at *p* ≤ 0.05; black letters for controls, grey letters for chilled plants.

Dry matter (DM), changed over time in chilled and non-chilled sage cultivars (Figure 1). The content of dry matter was significantly higher for both chilled cultivars as compared to the non-treated control; however, only for ‘Icterina’, on the 7th day, the DM of the chilled and control plants were statistically similar. Such a decrease in DM content caused by temperature was confirmed at the *p* ≤ 0.001 level (Table 1). The leaves of chilled sage lost more water than the control which probably could be attributed to disturbances in stomata functioning and turgor pressure in leaves due to an increase in cell membrane permeability and electrolyte leakage from cells [22]. The rapid decline in the ability of roots to absorb water and transport it to the shoots, as affected by chilling temperatures, was also referred [23,24]. On the other hand, Rodríguez et al. [25] described a significantly higher percentage of dry matter in *Brassica oleracea* plants grown under control than under chilling conditions. The highest differences in DM content due to chilling were found on the 10th day for ‘Icterina’ and on the 14th day for ‘Purpurascens (up to 1.90% FM and 2.51% FM, respectively). The dry matter changed in ‘Icterina’ chilled and non-chilled plants in the following days of chilling, but no general trend of these changes could be shown. The chilled ‘Purpurascens’ responded to low temperature through a decrease of DM on the 10th day, but later a constant increase was observed as a result of acclimatization processes. On the 7th day, the DM content of the control ‘Purpurascens’ plants dropped to a level that was sustained throughout the sampling period. The lower content of dry matter in ‘Purpurascens’ than in ‘Icterina’ was statistically confirmed at *p* ≤ 0.001 (Table 2).

**Figure 2:**
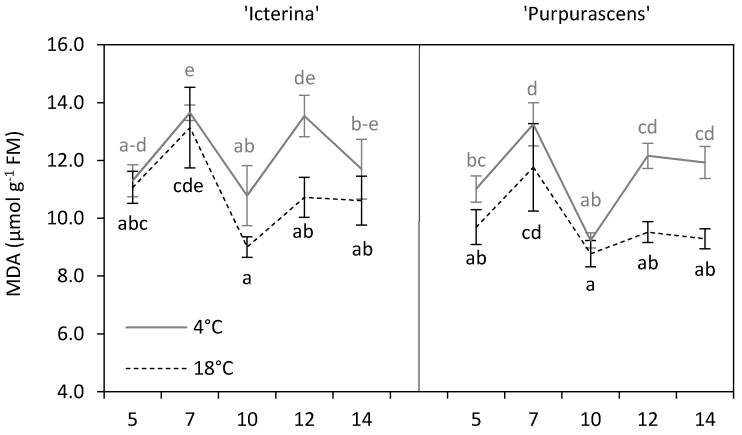
Malondialdehyde (MDA) content of ‘Icterina’ and ‘Purpurascens’ sage cultivars at different time points. Control plants (black dashed line) were grown at 18 °C, and chilled plants (grey line) were treated at 4 °C. Each graph is labeled number of the day (days from the start of chilling). Error bars represent ± standard deviation (*n* = 3). Different letters for the particular sage cultivars indicate significant differences in MDA content as affected by the chilling temperature and sampling time according to the Tukey’s HSD test at *p* ≤ 0.05; black letters for controls, grey letters for chilled plants.

An increase in malondialdehyde (MDA) content caused by chilling stress is associated with the stress-induced peroxidation of unsaturated fatty acids in phospholipids [7,15]. During the first 10 days of chilling, fluctuations in MDA content, parallel in chilled and control plants were observed for both cultivars (Figure 2). MDA reflects cellular damage caused by different stress factors, but the dynamic changes in its content need additional explanation. In the present experiment, we found a significant increase of MDA, up to 26%, in chilled ‘Icterina’ plants in comparison to control ones on the 12th day of chilling, but on the 14th day, MDA levels were similar between treatments. Higher content of MDA due to chilling was found in ‘Purpurascens’ cultivar on the 12th and 14th day, and differences reached in up to 28%. The analysis of the main effects (*p* ≤ 0.001) proved that low temperature generally increased the content of malondialdehyde in both sage cultivars (Table 1). Preliminary results did not confirm such a relationship in sage [26]; however, Sivaci et al. [17] described an increase in MDA content in chilled pepino plants in comparison to the control. Overall, our findings showed that overproduction of MDA occurred on the 12–14th day of chilling treatment. The MDA content, on average, was higher in ‘Icterina’ than in ‘Purpurascens’ at *p* ≤ 0.05 (Table 2). Xu et al. [20] showed that the MDA concentration in two tobacco cultivars (chill-tolerant and chill-sensitive) increased under chilling stress. On the other hand, Huang and Guo [8] observed that MDA content in chill-tolerant rice cultivar retained lower level in comparison to control, while that of the chill-sensitive cultivar increased rapidly with treatment; thus, variations between cultivars occurred in this respect. The changes in MDA noted for both sage cultivars in the control and stress conditions indicated that MDA content reflected chilling-induced cellular damage of leaf cells which appeared mostly after more than 10 days of chilling. The lack of differences in MDA content between control and chilled ‘Icterina’ after two weeks of treatment pointed to the more efficient acclimatization of this cultivar.

**Figure 3:**
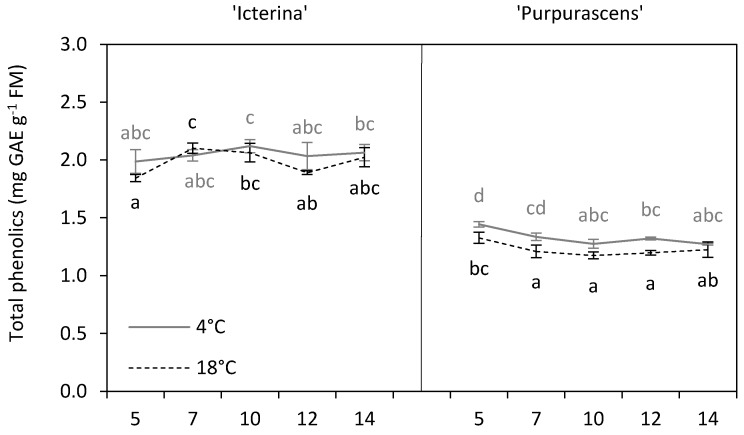
Total phenolics content of ‘Icterina’ and ‘Purpurascens’ sage cultivars at different time points. Control plants (black dashed line) were grown at 18 °C, and chilled plants (grey line) were treated at 4 °C. Each graph is labeled with the number of the day (days from the start of chilling). Error bars represent ± standard deviation (*n* = 3). Different letters for the particular sage cultivar indicate significant differences in total phenolics content as affected by the chilling temperature and sampling time according to the Tukey’s HSD test at *p* ≤ 0.05; black letters for controls, grey letters for chilled plants.

Total phenolics content (TPC) remained unchanged due to chilling in ‘Icterina’ plants for 14 days, as was shown by the analysis of the effect of the interaction of temperature and time (Figure 3). Some changes in TPC in control ‘Icterina’ plants occurred, an increase between the 5th and 7th or 10th day was observed, but the final level of these compounds was similar to the initial value. We found a higher content of total phenolics in chilled ‘Purpurascens’ plants on the 5th, 7th, and 12th day as compared to non-chilled controls. In chilled ‘Purpurascens’, the TPC decreased in time, while for control plants, first, a reduction was observed but later, the final level of total phenolics was similar to that at the beginning of the sampling period. The main effect of temperature were significant and generally showed an increase in TPC as affected by chilling as 3.5% (‘Icterina’, *p* ≤ 0.05) or 8.1% (‘Purpurascens’, *p* ≤ 0.001) (Figure 3, Table 1). This is inconsistent with our earlier results on sage, which pointed to non-significant changes in TPC due to chilling, but the data were averaged for six genotypes [26]. The non-enzymatic antioxidant components of the plants’ defense system include phenolic compounds, and their level is reported to alter in response to various stresses [7]. Oh et al. [9] observed that the total phenolics content of lettuce plants increased in response to chilling temperature, which is similar to general observations made in the present study. Kalisz et al. [15] found a differential response of basil cultivars to chilling regarding TPC because only one cultivar out of the six showed an increase in these compounds. This suggested that the TPC of individual cultivars could be shaped differently by stress stimuli. The mean values for the total phenolics content in sage cultivars were separated at *p* ≤ 0.001 (Table 2), and more phenolic compounds were found in ‘Icterina’ compared to ‘Purpurascens’. Habán et al. [27] found more rosmarinic acid, an ester of caffeic acid and 3,4-dihydroxyphenyllactic acid, in ‘Icterina’ sage than in ‘Purpurascens’ which clearly showed smaller content of phenolic compounds in ‘Purpurascens’ plants. To sum up, it has been demonstrated that total phenolics levels could be increased by chilling [17,28]. This was confirmed for both sage cultivars judging by the main effects analysis; however, in our opinion, the genetic factor was the most important determinant in this respect.

**Figure 4:**
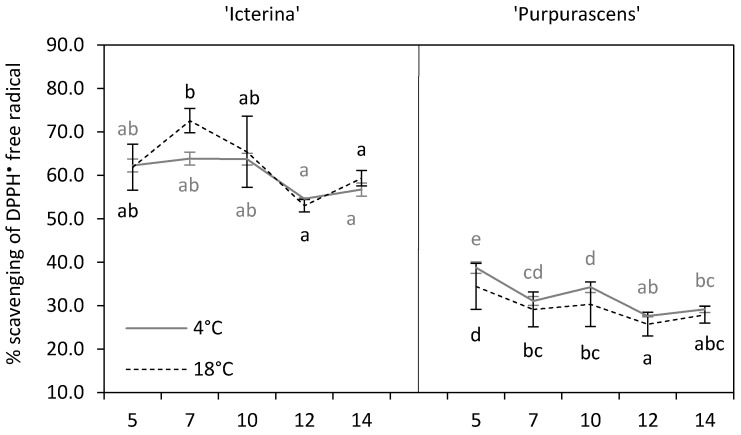
Total antioxidants capacity of ‘Icterina’ and ‘Purpurascens’ sage cultivars against 2,2-diphenyl-1-picrylhydrazyl (DPPH^•^) at different time points. Control plants (black dashed line) were grown at 18 °C, and chilled plants (grey line) were treated at 4 °C. Each graph is labeled with the number of the day (days from starting chilling). Error bars represent ± standard deviation (*n* = 3). Different letters for the particular sage cultivar indicate significant differences in DPPH^•^ scavenging activity as affected by the chilling temperature and sampling time according to the Tukey’s HSD test at *p* ≤ 0.05; black letters for the control, grey letters for the chilled plants.

Efficient scavenging of reactive oxygen species (ROS) produced during various environmental stresses requires the action of several non-enzymatic, as well as enzymatic antioxidants [7]. The measure of non-enzymatic compounds activity could be done through the 2,2-diphenyl-1-picrylhydrazyl (DPPH^•^) free radical scavenging assay [29,30]. The total antioxidant capacity (TAC) of plants could be correlated to phenolic compounds content [15]. Such a positive correlation (*r* = 0.97, *p* ≤ 0.001) was also found in the present study. There was no significant effect of chilling in ‘Icterina’ plants on DPPH^•^ free radical scavenging during subsequent sampling dates, while higher total antioxidants capacity was found for ‘Purpurascens’ sage on the 5th and 10th day as compared to the control (Figure 4). Non-significant alterations in TAC for ‘Icterina’ (*p* > 0.05) and significant alterations for ‘Purpurascens’ plants (*p* ≤ 0.001) due to the low-temperature treatment were confirmed by statistical analysis of the main effects (Table 1). We observed a decrease in the activity of the scavenging of DPPH^•^ radicals in control ‘Icterina’ between the 7th and the 12–14th day, while there were no changes observed over time for chilled plants. A gradual decrease in this activity was found in ‘Purpurascens’ plants. Analysis of the main effects showed that ‘Purpurascens’ had lower (of about 50%) total antioxidant capacity in comparison to ‘Icterina’ (*p* ≤ 0.001). Preliminary research on various sage cultivars also showed similar genotypic dependencies [26]. Pop et al. [30] observed that higher scavenging activity against DPPH^•^ radical was noted for ‘Icterina’ than ‘Purpurascens’ sage, but this difference was much smaller than that reported in our study and reached only 1%.

**Figure 5:**
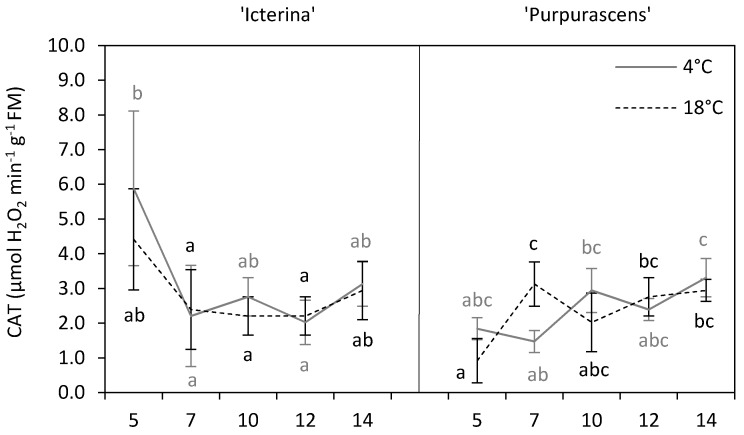
Catalase activity of ‘Icterina’ and ‘Purpurascens’ sage cultivars at different time points. Control plants (black dashed line) were grown at 18 °C, and chilled plants (grey line) were treated at 4 °C. Each graph is labeled with the number of the day (days from the start of chilling). Error bars represent ± standard deviation (*n* = 3). Different letters for the particular sage cultivar indicate significant differences in catalase activity as affected by the chilling temperature and sampling time according to the Tukey’s HSD test at *p* ≤ 0.05; black letters for the control, grey letters for the chilled plants.

Environmental stresses cause the overproduction of reactive oxygen species (ROS) [7]. Catalase (CAT) is responsible for catalyzing the dismutation of H_2_O_2_ into H_2_O and O_2_ in peroxisomes and mitochondria and thus completes the detoxification initiated by other enzymes [31]. In the present experiment, we did not find any significant effect of chilling on CAT activity (Figure 5), meaning that chilled and non-chilled plants of both sage cultivars did not differ at particular sampling points. The only exception was on the 7th day of chilling for the ‘Purpurascens’, where CAT activity was lower in chilled plants than in the control ones. The average of the effect of the temperature (main effects) did not show significant differences due to chilling treatment (*p* > 0.05) (Table 1). Apel and Hirt [1] reported that alterations in the balance of scavenging enzymes could cause up-regulation of some peroxidase enzymes when CAT activity was reduced in plants. However, some other researchers suggested that CAT activity increases in stress conditions along with an increase in the activity of other antioxidant enzymes [32]. Similar to our results, Kalisz et al. [15] found the changes in CAT activity to be negligible for almost all the tested genotypes of basil, with the exception of Thai basil, for which the activity of this enzyme dropped after chilling. Janda et al. [19] did not find significant changes in CAT activity in maize. In the present experiment, chilled ‘Icterina’ sage showed a decrease in CAT activity observed on the 7th and 12th day in comparison to the initial activity, while for control plants, the changes were small in the course of time. No significant changes in CAT activity level during time were noted for chilled ‘Purpurascens’ sage, while this activity increased in control plants between the 5th and 7th day, and finally was higher than at the beginning of the sampling period. Generally, ‘Icterina’ was a cultivar with a higher activity of this enzyme in comparison to ‘Purpurascens’ (Table 2, *p* ≤ 0.05). Differential responses to chilling of CAT activity in rice cultivars, differing in sensitivity to low temperature, was observed by Guo et al. [33], and was shown in maize inbred lines and hybrids by Janda et al. [19].

**Figure 6:**
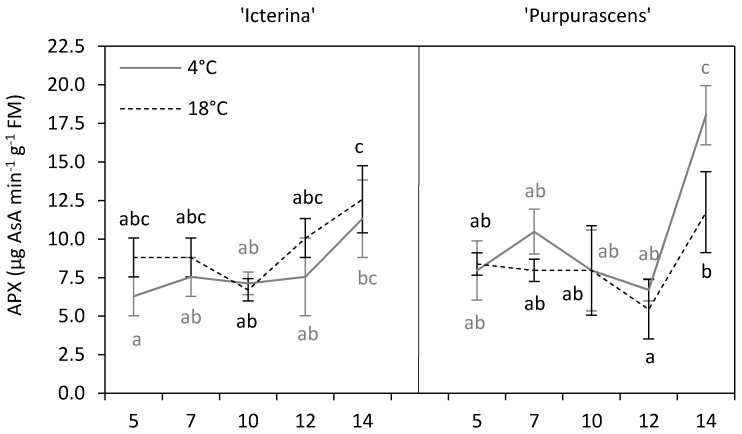
Ascorbate peroxidase activity of ‘Icterina’ and ‘Purpurascens’ sage cultivars at different time points. Control plants (black dashed line) were grown at 18 °C, and chilled plants (grey line) were treated at 4 °C. Each graph is labeled with the number of the day (days from the start of chilling). Error bars represent ± standard deviation (*n* = 3). Different letters for the particular sage cultivar indicate significant differences in the ascorbate peroxidase activity as affected by the chilling temperature and sampling time according to the Tukey’s HSD test at *p* ≤ 0.05; black letters for the control, grey letters for the chilled plants.

Ascorbate peroxidase (APX) catalyzes the conversion of H_2_O_2_ into H_2_O, using ascorbate as a specific electron donor [34]. It has been demonstrated that APX activity generally increases in plants in response to environmental stress [32,35]. In the present experiment, we did not observe substantial effects of chilling on APX activity at the particular time points (Figure 6). Only the ‘Purpurascens’ responded to chilling on the 14th day when significant increases in APX activity was observed, and at that time, the difference between the chilled and non-chilled plants was very high, reaching 53.6% (Table 1, *p* ≤ 0.05). It is interesting that APX activity was often slightly lower in chilled ‘Icterina’ plants in comparison to the control. However, an analysis of the main effects proved significant elevation in APX activity due to low-temperature treatment (Table 1, *p* ≤ 0.05). Duan et al. [36] found an increase of APX activity in tomato plants exposed to chilling temperature, and similar results were obtained by Guo et al. [37] for pepper seedlings. We found only a few differences in APX activity among the particular sampling points, the most pronounced was an increase observed for the ‘Purpurascens’ between the 12th and 14th day, and chilled plants had the highest APX activity after two weeks of low-temperature treatment. Chilled ‘Icterina’ also had higher APX activity at the end of chilling treatment in comparison to the initial level, with no substantial changes between treatments. We did not find any significant differences between sage cultivars (Table 2, *p* > 0.05), while genotypic diversity in the response of peroxidase activity to chilling was described by Kalisz et al. [15] for basil, Janda et al. [19] for maize plants, and Xu et al. [20] for tobacco.

**Figure 7:**
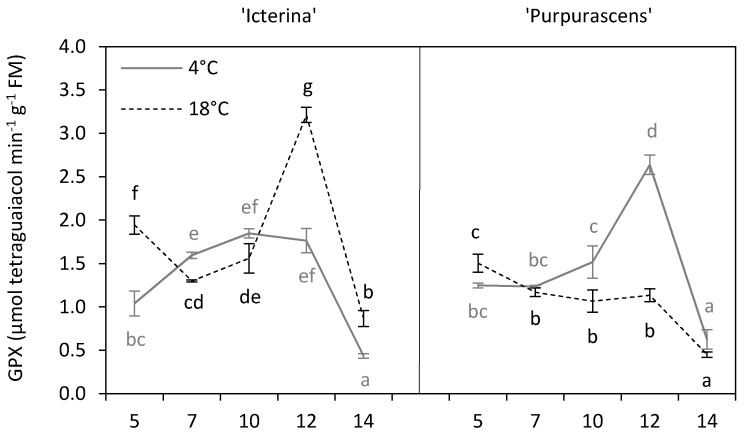
Guaiacol peroxidase activity of ‘Icterina’ and ‘Purpurascens’ sage cultivars at different time points. Control plants (black dashed line) were grown at 18 °C, and chilled plants (grey line) were treated at 4 °C. Each graph is labeled with the number of the day (days from the start of chilling). Error bars represent ± standard deviation (*n* = 3). Different letters for the particular sage cultivar indicate significant differences in guaiacol peroxidase activity as affected by the chilling temperature and sampling time according to the Tukey’s HSD test at *p* ≤ 0.05; black letters for the control, grey letters for the chilled plants.

The pattern of changes in guaiacol peroxidase (GPX) activity over time, an enzyme that oxidizes aromatic electron donors such as guaiacol and pyrogallol at the expense of H_2_O_2_ [7], was quite different in comparison to APX activity (Figure 7). Chilled ‘Icterina’ showed a higher activity of GPX in comparison to the control only on the 7th day of treatment, while on the 5th day and 12–14th days, GPX activity level was much higher for control plants. In the case of chilled ‘Purpurascens’, we observed a significant increase in GPX activity on the 10th and 12th days of treatment, after that its activity decreased and it became similar to that of control. The analysis of the main effects showed that chilling caused a decrease in GPX activity in ‘Icterina’ (*p* ≤ 0.001), but an increase in ‘Purpurascens’ (*p* ≤ 0.001). According to Yadegari et al. [14], soybean plants subjected to chilling responded with a higher activity of guaiacol peroxidase than control ones. The changes in GPX activity during the chilling treatment were slightly different between the sage genotypes (Figure 7); however, an analysis of main effects did not show that the tested cultivars differed significantly (Table 2, *p* > 0.05). Thus an increase in GPX activity could be the result of oxidative stress, but the time of activation could be related to the plant genotype. This was confirmed by Janda et al. [19] who found an increase in GPX activity only in one inbred maize line among nine genotypes.

## 3. Materials and Methods

### 3.1. Plant Material and Treatment Conditions

Sage (*Salvia officinalis* L.) plants were raised from cuttings in 1 dm^3^ pots filled with peat substrate. Two sage cultivars were used: ‘Icterina’ obtained from the Ogród Łobzów nursery (Kraków, Poland) and ‘Purpurascens’ from the Školky Litomyšl nursery (Litomyšl, Czech Republic). ‘Icterina’ is a sub-shrub with greyish leaves, with yellow and pale green variegation, while ‘Purpurascens’ is a sub-shrub with purple leaves maturing to silvery-green. Plants were grown in the same microclimatic conditions up to chilling. When 8–10 leaves were developed, half of the plants of each cultivar were subjected to 4 °C for two weeks, while non-chilled plants (18 °C) were considered controls. In both growing chambers, the length of the day time was set to 12 h, the canopy level irradiance intensity in the photosynthetically active radiation range (400–700 nm) of approximately 200 μmol m^−2^ s^−1^ was provided by OSRAM BIOLUX (Munich, Germany) T8 L-36-965 tubular fluorescent lamps and the relative humidity was 75% ca. Plants were randomly arranged in the growing chamber. They were sampled five-fold, at two three-day intervals, starting from the 5th till the 14th day of temperature treatment. For each sampling date, 28 plants were taken and then all were fully developed, healthy leaves were always cut and submitted for laboratory analysis.

### 3.2. Dry Matter

Dry matter (DM) was determined according to the standard gravimetric method. Samples were weighed with a Sartorius A120S balance (Sartorius AG, Göttingen, Germany) and dried at 65 °C until a constant weight was achieved. The difference in weight was calculated and expressed as a fresh matter (FM) percentage.

### 3.3. Malondialdehyde

The malondialdehyde (MDA) content was measured by the thiobarbituric acid method, according to Dhindsa and Matowe [38]. Plant samples were homogenized in 0.1% trichloroacetic acid (TCA) and centrifuged at 13,968× *g* for 15 min. The reaction mixture consisted of the supernatant, 0.1 M K-phosphate buffer (pH 7.6) and 0.5% thiobarbituric acid dissolved in 20% TCA. The absorbance of the supernatant was read at 532 nm and corrected for non-specific turbidity by subtracting the absorbance at 600 nm (UV-VIS Helios Beta spectrophotometer, Waltham, MA, USA). The amount of MDA was calculated from the difference in absorbance at these wavelengths, using a molar absorbance coefficient of 155 mM^−1^ cm^−1^. TCA (0.1%) was used as a blank.

### 3.4. Total Phenolics

Total phenolics content (TPC) was estimated using the modified Folin–Ciocalteu colorimetric method [39]. Fresh plant material (2.0 g) was mixed with 10 cm^3^ of 80% methanol; then samples were centrifuged (3492× *g*, 10 min). Plant extracts (0.1 cm^3^) were mixed with 2 cm^3^ of sodium carbonate, after the next 2 min, 0.1 cm^3^ Folin–Ciocalteu’s reagent, mixed with deionized water (1:1 *v*/*v*), was added to the test tubes. The absorbance of the resulting blue color was measured at 750 nm using the UV-VIS Helios Beta spectrophotometer against a reference solution. The results are expressed as gallic acid equivalents (GAE); milligrams GAE per 1 g FM.

### 3.5. DPPH^•^ Radical Scavenging Activity

The total antioxidant activity (TAC) was determined according to the method of Bartosz [40] using the artificial radical 2,2-diphenyl-1-picrylhydrazyl (DPPH^•^). A mixture of fresh plant material (2.0 g) was grounded and centrifuged (3492× *g*, 10 min) with 80% methanol. Test tubes contained 0.1 cm^3^ supernatant and 4.9 cm^3^ of 0.1 mM DPPH dissolved in 80% methanol. The mixture was incubated for 15 min in the dark and at 20–22 °C, the absorbance was measured using the UV-VIS Helios Beta spectrophotometer at 517 nm. The antioxidant activity was calculated as DPPH [%] = [(A_0_ − A_1_)/A_0_] × 100; where A_0_ and A_1_ are the absorbance of the reference and test solutions, respectively.

### 3.6. Antioxidant Enzyme Assays

Catalase (CAT, EC 1.11.1.6) activity was determined by measuring the disappearance of H_2_O_2_ spectrophotometrically at 240 nm (UV-VIS Helios Beta spectrophotometer) as described by Aebi [41]. Plant material (2.5 g of fresh leaves) was ground in an ice bath with 20 cm^3^ 0.05 M potassium phosphate buffer and centrifuged (3492× *g*, 15 min, 4 °C). Test tubes contained 1.8 cm^3^ 0.05 M phosphate buffer (pH 7.0) and 1.0 cm^3^ 0.05% H_2_O_2_ solution in 0.05 M potassium phosphate buffer (pH 7.0). Supernatant (0.2 cm^3^) was added and the absorbance was read every minute for 5 min, and the decrease in absorbance against a blank was measured. The specific activity was calculated and presented as micromoles of H_2_O_2_ per 1 min per 1 g FM.

The initial procedure for peroxidase enzymes extraction: 2 g of leaf samples were homogenized in 50 mM potassium phosphate buffer (pH 7.0) containing 1 mM ethylene diamine tetraacetic acid (EDTA), 1% soluble polyvinyl pyrrolidone (PVP), and 1 mM phenylmethylsulfonyl fluoride (PMSF). All extraction steps were carried out on the ice at 4 °C. The mixture was centrifuged at 13,968× *g* for 15 min, and the supernatant was used for enzyme activity assays. Ascorbate peroxidase (APX, EC 1.11.1.11) activity was assayed according to the method of Nakano and Asada [42]. The reaction mixture contained 50 mM potassium phosphate buffer (pH 7.0), 0.5 mM ascorbate, 0.1 mM H_2_O_2_, and 0.15 cm^3^ of the enzyme extract. The hydrogen peroxide-dependent oxidation of ascorbate (AsA) was followed by a decrease in the absorbance at 290 nm (UV-VIS Helios Beta spectrophotometer) in every minute for 5 min (ε = 2.8 mM^−1^ cm^−1^). APX activity was expressed as µg AsA min^−1^ g^−1^ FM. Guaiacol was used as a substrate to evaluate the activity of guaiacol peroxidase (GPX, EC 1.11.1.7) [43]. The reaction mixture consisted of 50 mM phosphorus buffer (pH 7.0), hydrogen peroxide (1%), and guaiacol (4%). The reaction was started by adding 0.2 cm^3^ of enzyme extract to the reaction mixture at 25 °C. The increase in absorbance at 470 nm due to guaiacol oxidation was recorded using UV-VIS Helios Beta spectrophotometer over a period of 3 min (ε = 26.6 mM^−1^ cm^−1^). GPX activity was presented as µmol tetraguaiacol min^−1^ g^−1^ FM.

### 3.7. Statistical Analysis

All obtained data were analyzed statistically in Statistica 13.3 (Tibco Software Inc., Palo Alto, CA, USA). Analysis of variance (ANOVA) followed by Tukey’s HSD tests was used at *p* ≤ 0.05 (*), *p* ≤ 0.01 (**), and *p* ≤ 0.001 (***). Two-way statistically significant differences between temperature (T), sampling time (S), and T × S interactions were determined. Homogeneous groups of means were determined with the Tukey’s HSD test at the significance level 0.05. The changes in the chemical constituents’ content or their activity depending on the sage cultivar were screened by using the t-test as mentioned before the probability levels. Pearson correlation analysis was performed between antioxidant activity and total phenolics content using Statistica 13.3 (*n* = 20).

## 4. Conclusions

In conclusion, two sage cultivars had different results in response to low temperature; the treatment activated antioxidant mechanisms, which worked differently. The ‘Purpurascens’ sage showed much higher flexibility of antioxidant mechanisms, including enzymatic and non-enzymatic components, in comparison to ‘Icterina’ plants. The activities of antioxidant peroxidases (APX and GPX) increased in chilled plants of ‘Purpurascens’, finally, APX activity stayed the highest throughout the chilling period, but GPX activity decreased to that of the control. The chilling also caused an increase in the total phenolics compounds concentration and antioxidant capacity of ‘Purpurascens’ plants at specific time points as compared to control plants. However, ‘Icterina’ had significantly higher total phenolics content and higher DPPH^•^ scavenging activity in comparison to ‘Purpurascens’. We observed a similar level of MDA at the end of chilling (14th day) for ‘Icterina, and higher levels of MDA on the 12th and 14th day in ‘Purpurascens’ plants subjected to chilling. Therefore, we conclude that oxidative stress existed in ‘Purpurascens’ sage at that time, despite the operation of the antioxidant mechanisms, while antioxidants of ‘Icterina’ deactivated ROS more effectively, as evidenced by the reduction of lipid peroxidation. Data of the present experiment substantiated that such genotypic differences should be considered in breeding programs, especially nowadays when stress acclimatization is a crucial characteristic for producers.

## Figures and Tables

**Table 1:** Results of the Tukey’s HSD test for the analyzed parameters in sage cultivars as influenced by temperature (T: 4 °C, chilling; 18 °C, control), sampling time (S: 5; 7; 10; 12, and 14 days from the start of chilling) and T × S interaction, *n* = 3.

Parameter	Source of Variation					
	Icterina			Purpurascens		
	T	S	T × S	T	S	T × S
Dry matter	***	***	***	***	***	***
Malondialdehyde	***	***	***	***	***	***
Total phenolics	*	*	**	***	***	***
Antioxidant capacity	ns	***	***	***	***	***
Catalase	ns	**	*	ns	***	**
Ascorbate peroxidase	*	***	**	*	***	***
Guaiacol peroxidase	***	***	***	***	***	***

Level of significance: * *p* ≤ 0.05; ** *p* ≤ 0.01; *** *p* ≤ 0.001; ns—not significant.

**Table 2:** Changes in the content or activity of the chemical constituents as depending on sage cultivar with the results of the t-test separation into homogenous groups at *p* ≤ 0.05 with the means for sage cultivars, *n* = 30.

Parameter	Unit	Icterina	Purpurascens	Significance
Dry matter	% FM	17.41 ^b^	14.97 ^a^	***
Malondialdehyde	µmol g^−1^ FM	11.55 ^b^	10.66 ^a^	*
Total phenolics	mg GAE g^−1^ FM	2.02 ^b^	1.28 ^a^	***
Antioxidant capacity	% scavenging of DPPH^•^	61.36 ^b^	30.86 ^a^	***
Catalase	µmol H_2_O_2_ min^−1^ g^−1^ FM	3.02 ^b^	2.37 ^a^	*
Ascorbate peroxidase	µg AsA min^−1^ g^−1^ FM	8.68 ^a^	9.27 ^a^	ns
Guaiacol peroxidase	µmol tetraguaiacol min^−1^ g^−1^ FM	1.56 ^a^	1.26 ^a^	ns

Means within a row followed by different letters are significantly different at *p* ≤ 0.05 according to Tukey’s HSD test. Level of significance: * *p* ≤ 0.05; ** *p* ≤ 0.01; *** *p* ≤ 0.001; ns—not significant.
